# Diffuse large B-cell lymphoma microenvironment displays a predominant macrophage infiltrate marked by a strong inflammatory signature

**DOI:** 10.3389/fimmu.2023.1048567

**Published:** 2023-05-02

**Authors:** Leyre Serna, Peio Azcoaga, Manisha Brahmachary, Maria M. Caffarel, Mounia S. Braza

**Affiliations:** ^1^ Faculty of Science and Technology, Department of Biochemistry and Molecular Biology, University of the Basque Country (UPV/EHU), Leioa, Spain; ^2^ Oncology Department, Biodonostia Health Research Institute, San Sebastian, Spain; ^3^ Department of Oncological Sciences, Icahn School of Medicine at Mount Sinai, New York City, NY, United States; ^4^ Ikerbasque Basque Foundation for Science, Bilbao, Spain

**Keywords:** cancer, lymphoma, inflammation, inflammasome, immunology

## Abstract

Inflammasomes are cytosolic signaling hubs that promote the inflammatory response (i.e. an immune reaction to counteract threats in physiological conditions). Their potential role in lymphomagenesis remains to be elucidated. Depending on the context, innate immune cells, such as macrophages, may induce inflammation that contributes to the anti-tumor function; however, if uncontrolled, inflammation can promote cancer development. Here, we exploited bioinformatic tools, TCGA data, and tumor tissue samples from patients with diffuse large B-cell lymphoma (DLBCL), one of the most frequent non-Hodgkin lymphomas of B-cell origin, to investigate the distribution of the different immune cell subpopulations in DLBCL samples in order to characterize the immune landscape of their microenvironment. We found a clear prominence of macrophages in the DLBCL microenvironment. Particularly, the proportions of resting M0 and pro-inflammatory M1 macrophages were higher in DLBCL than spleen samples (controls). As each inflammasome has unique sensor activation and platform assembly mechanisms, we examined the expression of a large panel of inflammasome actors. We found that inflammasome components, cytokines and Toll-like receptors were upregulated in DLBCL samples, particularly in M0 and M1 macrophages, compared with controls. Moreover, their expression level was positively correlated with that of CD68 (a pan-macrophage marker). We confirmed the positive correlation between CD68 and IRF8 expression at the protein level in DLBCL tissue samples, where we observed increased infiltration of CD68- and IRF8-positive cells compared with normal lymph nodes. Altogether, our results highlight the inflammatory status of the DLBCL microenvironment orchestrated by macrophages. More work is needed to understand the complexity and potential therapeutic implications of inflammasomes in DLBCL.

## Introduction

Non-Hodgkin Lymphoma (NHL) describes a diverse group of lympho-proliferative disorders that includes most hematological malignancies (1). Diffuse Large B-cell Lymphoma (DLBCL) is one of the most common and aggressive B-cell NHLs (~35% of all B-cell NHLs worldwide) (2). Gene expression profiling studies have contributed to the definition of different DLBCL subgroups, including germinal center B-cell like DLBCL (GCB subgroup) and activated B-cell like DLBCL (ABC subgroup) that are characterized by constitutive activation of the nuclear factor kappa-light-chain-enhancer of activated B cells (NFKB) pathway (3). First-line rituximab (anti-CD20 monoclonal antibody) has significantly increased the complete response rate in patients with DLBCL; however, almost 30% of patients will relapse with poor clinical outcomes (4). Therefore, it is important to develop novel therapeutic approaches that are more efficient in the long term. To this aim, it is crucial to understand the immunological mechanisms underlying DLBCL development and progression.

In the tumor microenvironment (TME), immune cells play a central role in surveillance and crosstalk with the other cells. Importantly, an inflammatory microenvironment is strongly implicated in cancer development and progression. Macrophages are innate immune cells that play a major role in the initiation of inflammation, an immune reaction to different threats (e.g. microbial infections, allergies, cancer). However, chronic inflammation can also promote cancer development and malignant cell immune escape, thus contributing to tumor progression (5). In an exacerbated inflammatory context, macrophages might play important roles in the malignant cell-TME crosstalk and promote tumorigenesis (1, 6).

Previous studies investigated the different inflammatory cells in the DLBCL microenvironment to establish correlations with prognosis, stage-related tumor progression, and treatment outcome. Specifically, they showed that the presence of CD68-positive tumor-infiltrating macrophages correlates with poor prognosis in the aggressive B-cell NHL subtype (7, 8). Moreover, Guidolin et al. (9) quantitatively evaluated the morphological features and spatial patterns (the percentage and position of positive cells in the tissue) of inflammatory cells (including macrophages) in the DLBC microenvironment. They used a morphological approach based on the estimation of an uniformity index and a spatial statistic approach that involved calculating the Ripley’s K-function of the distances between cells (9). Their found a significantly higher immune infiltrate and uniform distribution in the more aggressive ABC subtype than in the GBC subtype (where immune cells were clustered). The only exception concerned the distribution of CD68-positive cells that was similar in both DLBCL subtypes. These features may greatly influence the interaction of the many different cell populations within the TME and affect the distribution of key factors that drive tissue growth (9–11).

Inflammasomes are cytosolic multiprotein complexes that mediate the initial inflammatory responses to danger signals. Inflammasomes are generally constituted of a sensor molecule connected to an inflammatory caspase through an adaptor protein. Their assembly can be triggered by a multitude of microbial and host-derived stimuli. Inflammasomes rely on a two-step activation model: priming (a necessary regulation to avoid over-activation) and activation (in which post-translational modifications of the primed sensor are induced, resulting in conformational changes). Once activated, inflammasomes drive the formation of gasdermin-D pores at the cell surface. This leads to the non-conventional secretion of interleukin (IL)-1β and IL−18 pro-inflammatory cytokines, IL-1α, high mobility group box 1 (HMGB1), alarmins, and ultimately to pyroptotic cell death. Inflammasomes are defined as ‘canonical’ when they include human caspase-1, and ‘non-canonical’ when they include human caspase-4 or caspase-5 (or their murine ortholog caspase-11) (12). Inflammasomes have beneficial roles (e.g. micro-organism clearance, anti-tumor function); however, their aberrant or excessive activation might contribute to cancer development by promoting a strong and chronic pro-inflammatory microenvironment. Therefore, we wanted to analyze the inflammasome and inflammatory cell landscape in the DLBCL microenvironment. To this aim, we exploited publicly available The Cancer Genome Atlas (TCGA) (for DLBCL samples) and GTEx (for control samples) data and bioinformatic tools to comprehensively investigate i) the immune cell infiltrate and the proportion of M0, M1 and M2 macrophages; ii) the impact on patient survival of the expression of different inflammatory factors; iii) the correlation between the expression levels of inflammasome components and of CD68 (a macrophage marker); and iv) the inflammatory state (cytokine and Toll-like receptor, TLR, expression) of the different macrophage subtypes and their enrichment in the TME of DLBCL samples. We validated some of our results by immunohistochemistry (IHC) analysis of DLBCL samples from an independent cohort where we showed increased expression of CD68 and IRF8 compared with control lymph node samples. Overall, our findings suggest that macrophages orchestrate a pro-inflammatory microenvironment in DLBCL.

## Materials and methods

To perform the bioinformatic analysis, we used a TCGA dataset that included 48 DLBCL samples. We analyzed their immune infiltrate and their gene expression profile using ImmuCellAI and GEPIA2, respectively. Of note, only 47 of these samples were included in GEPIA2 (the patients’ characteristics are listed in **Table S1**). We also used data on 337 spleen tissue samples (controls) from the GTEx database.

### ImmuCellAI

ImmuCellAI is a bioinformatic tool to predict the immune cell abundance in a sample. It was developed and is maintained by An-Yuan Guo’s laboratory (13), College of Life Science and Technology, HUST, China (guoay@hust.edu.cn, http://bioinfo.life.hust.edu.cn/web/ImmuCellAI/). It is a comprehensive resource that can be used for the systematic analysis of immune infiltrates in different cancer types. We used this tool and the GSCA/GSCALite integrated database to estimate the abundance of tumor-infiltrating immune cells in DLBCL samples.

### Gene expression profiling interactive analysis 2

GEPIA is an enhanced web server for large-scale expression profiling and interactive analysis (http://gepia.cancer-pku.cn/index.html). It is a valuable and highly cited resource for gene expression analysis based on tumor and normal samples from the TCGA and the GTEx databases. GEPIA (14) is an interactive web interface that includes 9,736 tumors and 8,587 normal samples from TCGA and GTEx projects to analyze gene expression by RNA sequencing. We used GEPIA2 to analyze the gene expression correlation for the available DLBCL samples in TCGA (n= 47), the overall survival significance of specific genes in DLBCL, the differential gene expression profile between DLBCL and normal spleen samples (n= 337), and to perform multiple deconvolution-based analyses. In 2021, GEPIA developed an extension with multiple deconvolution-based analyses. Each sample is deconvoluted in TCGA/GTEx with the bioinformatics tools CIBERSORT, EPIC and quanTIseq. Based on the inferred cell proportions in each bulk-RNA sample, various downstream analyses can be performed, such as proportion, correlation, sub-expression and survival (15).

### Gene set enrichment analysis

We performed GSEA using the knowledge-based approach for interpreting genome-wide expression profiles previously described by Subramanian A et al. (16). We compiled and manually curated the 80-gene inflammatory signature (**Table S2** and **Figure S1F**) from literature data. We analyzed this gene set against the pre-ranked gene list of differentially expressed genes in DLBCL versus spleen samples. We considered a false discovery rate (FDR) cut-off <0.05 as significant.

### Tissue microarrays and immunohistochemistry

For the IHC analysis, we used 192 lymphoma samples (different subtypes) included in a tissue microarray (TMA): 118 DLBCL, 3 Burkitt-like lymphoma, 5 follicular lymphoma, 1 mantle cell lymphoma, 4 plasma cell lymphoma, 7 anaplastic large cell lymphoma, 22 T-cell lymphoma, 4 angioimmunoblastic T-cell lymphoma, 12 Hodgkin’s lymphoma, and 16 lymph node (control) samples. One single core per sample was included in the TMA and the patients’ characteristics are summarized in **Table S3**. We used seven additional normal lymphoid tissue types (appendix, bone marrow, lymph node, placenta, spleen, thymus, tonsil), included in another TMA in duplicate, as controls. Specifically, we considered normal lymph nodes (n=18) as negative controls. We used the other six lymphoid tissue types as positive controls to confirm staining, but did not quantify staining because each tissue was represented by only one sample (in duplicate) and thus not enough for statistical analysis. We excluded few damaged cores (n= 20). For IHC, after heat-induced epitope retrieval, we incubated TMA sections with anti-CD68 (Abcam, ab201340, UK) and anti-IRF8 (Abcam, ab207418, UK) antibodies at 4 °C overnight, followed by biotin-streptavidin horseradish peroxidase-conjugated secondary antibodies and 3,3′ -diaminobenzidine. We used a light microscope (Nikon 80i) to count the number of positive cells per core. We then calculated the histoscore for each sample by summing the (a) score given in function of the absolute count of positive cells per core (0 for ≤5, 1 for 6-50, 2 for 51-100, 3 for 101-150, and 4 for ≥151) and the (b) staining intensity (0 for no staining, 1 for light brown-yellow, 2 for brown-yellow, 3 for dark brown-yellow). Thus, histoscore = a + b.

### Statistical analysis

We described the results using P-values, fold changes, ranks and correlation coefficients. We considered significant p-values ≤0.05. We performed the pair-wise gene expression correlation analysis of TCGA and GTEx expression data with the Pearson’s, Spearman’s and Kendall’s correlation coefficients. GEPIA uses the non-log scale for calculation and the log-scale axis for visualization. We estimated the survival contribution of specific genes related to inflammation in DLBCL with the Mantel–Cox test that displays the results as log10 hazard ratios (HR). We compared the cell type proportions and differential gene expressions (proportion analysis and sub-expression analysis) between groups with the one-way ANOVA test. For the GSEA, we used a FDR cut-off ≤0.05. We presented the IHC results as the mean ± SD and compared them with the 2-tailed unpaired Student’s *t*-test or the Mann-Whitney test.

## Results

### Macrophages are the most abundant immune infiltrate in DLBCL

**Figure 1:**
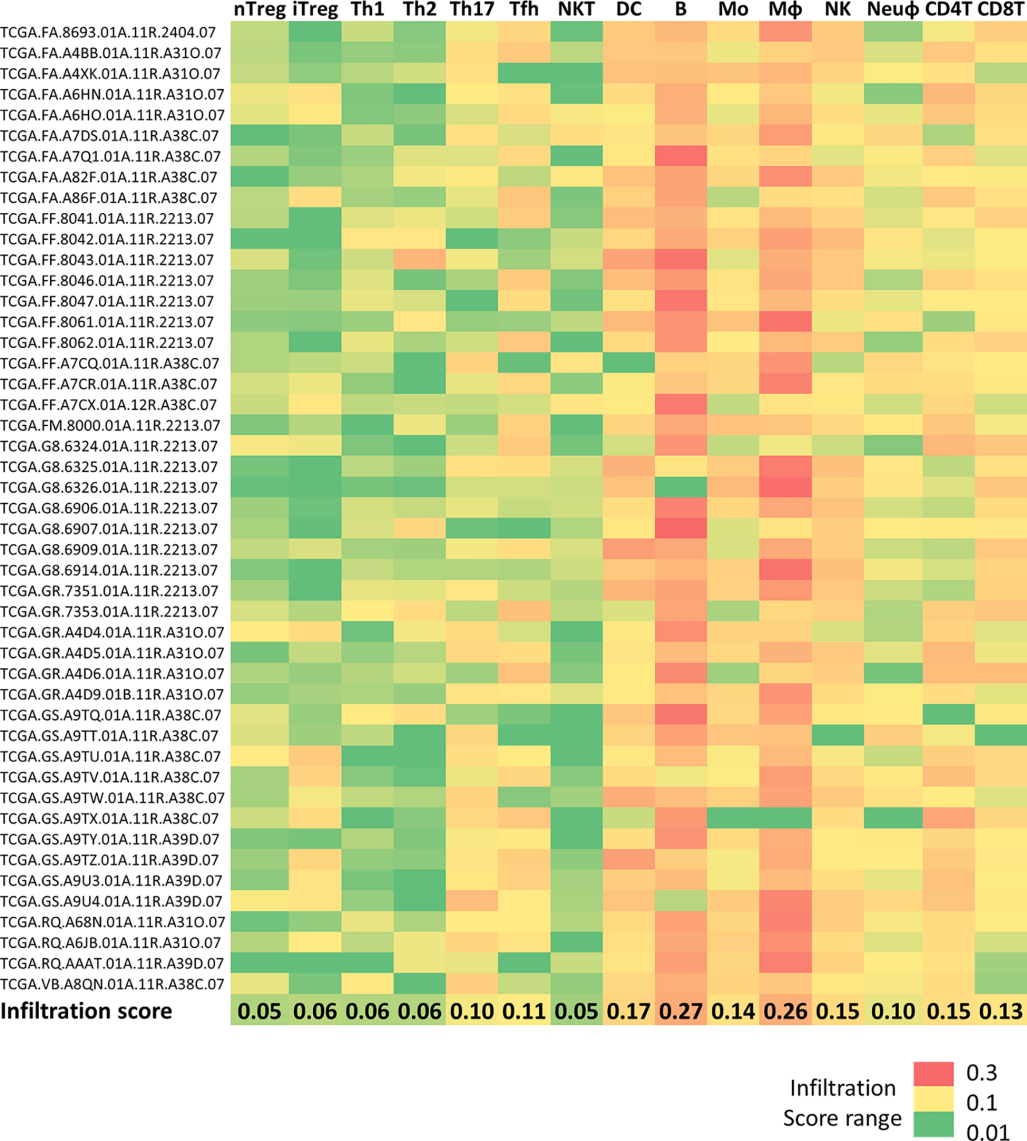
Immune cell infiltration in DLBCL and spleen samples Heatmap showing the infiltration scores of the indicated immune cell subtypes in 48 DLBCL samples from the TCGA expression database. The last line shows the mean infiltration score for each cell type. Green to red color: low to high infiltration score. Raw data were extracted from the ImmuCellAI database, analyzed, and converted into the heatmap. nTreg, natural regulatory T cells; iTreg, induced regulatory T cells; Th1, T helper 1 cells; Th2, T helper 2 cells; Th17, T helper 17 cells; Tfh, T follicular helper cells; NKT, natural killer T cells; DC, dendritic cells; B, B cells; MO, monocytes; MФ, macrophages; NK, natural killer cells; Neuφ, neutrophils; CD4T, CD4 T cells; CD8T, CD8 T cells.

First, to investigate the DLBCL immune microenvironment, we used data on 47 DLBCL samples from the TCGA database and on 337 spleen samples (secondary lymphoid organ, as healthy tissue control). We exploited the ImmuCellAI tool to extract the infiltration scores for the different immune cell subtypes that we transformed into a heat map to easily display and compare their proportions (regulatory T cells, T helper cells, natural killer T cells, dendritic cells, B cells, monocytes, macrophages, natural killer cells, neutrophils, CD4 T and CD8 T cells). The highest mean infiltration score (0.27) was for B cells (the cells from which DLBCL originate), followed by macrophages (mean infiltration score = 0.26). Conversely, natural killer T cells were the least abundant immune population in the TME (**Figure 1**).

We then used the deconvolution analysis tool of the GEPIA2 platform, and specifically the cell proportion analysis tool, to confirm and visualize the proportion of each immune cell type (CD4 T and CD8 T cells, regulatory T cells, natural killer cells, neutrophils, dendritic cells). This analysis confirmed the heat map results showing a predominant proportion of macrophages in DLBCL samples compared with controls (**Figure S1A** and **Table S4**). Although to a lesser extent, the CD4 T-cell proportion also was higher in DLBCL than in control samples (tumor/spleen fold change: 1.3e+01, p ≤1.0e-6) (**Figure S1A** and **Table S4**). Conversely, we did not find any significant difference in the proportion of the other immune cell populations. Using this GEPIA2 interactive tool, we also obtained more detailed information on the distribution of the three different macrophage subtypes (resting M0, and polarized M1 and M2 macrophages) in DLBCL samples compared with controls. Specifically, the proportions of M0 and M1 macrophages were higher in DLBCL than in control samples (tumor/spleen fold change: 9.4e+01, p =5.5e-14 and 2.9e+01, p =1.1e-10, respectively). Conversely, the proportion of M2 cells (immunosuppressive and anti-inflammatory macrophages) was significantly higher in control than in DLBCL samples (tumor/spleen fold change: 2.2e-01, p ≤1.7e-5) (**Figure S1B** and **Table S4**). M0 macrophages are differentiated unpolarized macrophages that can be reprogrammed into polarized M1 macrophages (a pro‐inflammatory phenotype) or M2 macrophages (an immunosuppressive cell type) (17). Therefore, our findings suggest a preferential polarization of M0 macrophages into M1 macrophages in DLBCL samples compared with controls. The high proportion of M0 and M1 macrophages in DLBCL samples suggests a shift towards a pro-inflammatory state.

### The expression levels of inflammasome components and of the macrophage marker CD68 are correlated in DLBCL

**Figure 2:**
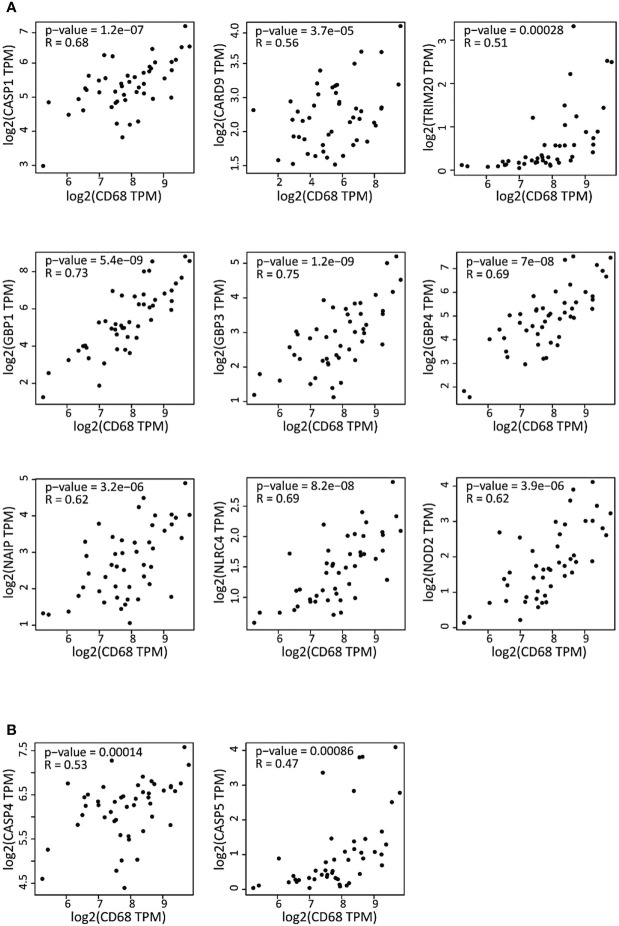
Correlation of the expression of inflammasome genes and of the CD68 macrophage marker in DLBCL samples **(A)** Correlation between the expression of the canonical inflammasome genes and *CD68* in 47 DLBCL samples (TCGA database). **(B)** Correlation between the expression of non-canonical inflammasome genes and *CD68* in the same DLBCL samples. Quantitative comparison based on the Pearson’s correlation coefficient **(R)**. Y-axis: log2 (TPM) of the inflammasome gene expression level, X-axis: log2 (TPM) of *CD68* expression level. TPM, transcript count per million reads.

As we observed a predominance of M1 pro-inflammatory macrophages in DLBCL, indicative of an elevated inflammatory state, we next examined the correlation between the gene expression of inflammasome components and of *CD68* (a marker of functionally active macrophages associated with altered gene expression within the tumor) (18). In DLBCL samples, *CD68* expression was significantly and positively correlated with the expression of the key inflammasome markers *CASP1*, *CARD9*, *TRIM20*, *GBP1*, *3* and *4*, *NAIP*, *NLRC4*, and *NOD2* (0.51≤ R ≤0.75; p ≤0.00028), most of which are part of the canonical inflammasome machinery (*GBP1*, *GBP3*, *GBP4*, *CASP1*, *NAIP*, *NLRC4*, and *NOD2*) (**Figure 2A**). In addition, *CD68* expression levels were significantly correlated only with the expression of the *CASP4* and *5* non-canonical inflammasome components (**Figure 2B**) (R ≤0.53; p ≤0.00086). Then, we asked whether the expression level of these inflammasome molecules might influence the survival of patients with DLBCL. Using the Mantel-Cox test, we found that some of them (*GBP3*, *IRF4*, *NAIP, NLRC4* and *NLRP1*) were negatively associated with survival (Log10 HR >0.0 and p ≤0.05), while others (*ALK, CASP5, GBP1, GBP4*and *NLRP3*) were positively associated with survival (Log10 HR <0.0 and p ≤0.05) (**Figure S1C**). Overall, these results indicate a strong correlation between the expression levels of the CD68 macrophage marker and inflammasome components. Conversely, the findings on the survival significance of these inflammatory factors were inconclusive.

### Inflammasome components are upregulated in M0 and M1 macrophages in DLBCL

**Figure 3:**
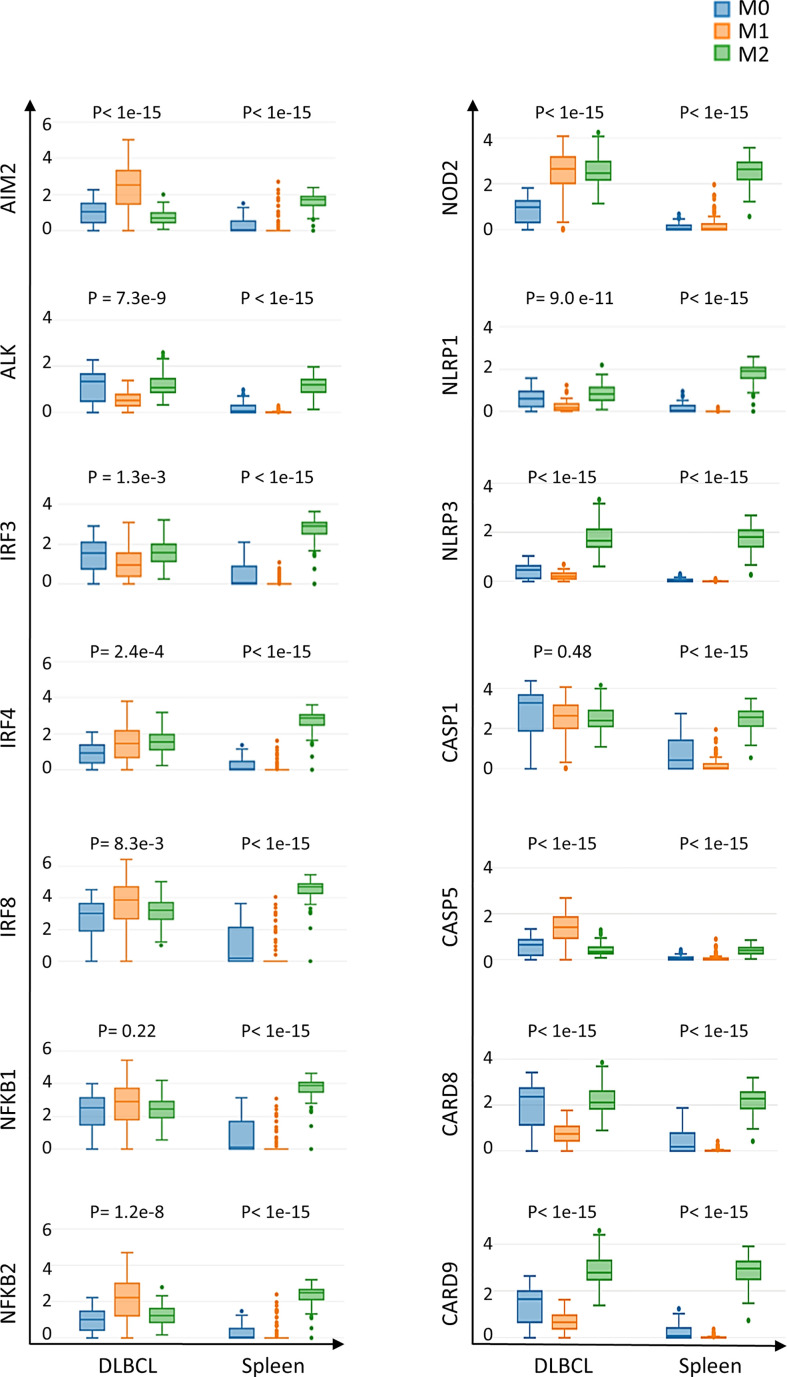
Differential expression of inflammasome components in M0, M1 and M2 macrophages in DLBCL and spleen samples. Comparison of the gene expression of key inflammasome molecules in M0, M1 and M2 macrophages in 47 DLBCL samples (TCGA database) and 337 spleen tissue samples (normal secondary lymphoid organ tissue; GTEx database) Y-axis: log (TPM+1) of the gene expression level. Additional data are in **Table 1**. Significance: P ≤0.05 (one-way ANOVA). TPM, transcript count per million reads. It is important to note that the results of the deconvolution analyses in **Figures 3**, **4** display comparisons according to the tissue type (DLBCL and spleen) and not the cell type (M0, M1 and M2).

The findings that the expression levels of inflammasome components and of the *CD68* macrophage marker are correlated and that not all macrophage subsets equally infiltrate DLBCL (**Figure S1A**) led us to precisely investigate the expression of inflammasome components in the different macrophage subpopulations (M0, M1 and M2) in DLBCL. We observed a significant upregulation of key inflammasome components (*AIM2*, *ALK*, *IRF3, 4* and *8*, *NFKB1* and *2*, *NOD2*, *NLRP1* and *3*, *CASP1* and *5*, *CARD8* and *9*) in M0 and M1 macrophages in DLBCL compared with spleen samples (tumor/spleen fold change: 5.8e+01 to 5.6e+04 for M0; 1.4e+02 to 3.9e+06 for M1, p ≤0.01, respectively; except for *NFKB1*: p= 0.22), but not in M2 macrophages (tumor/spleen fold change: 4.3e-04 to 2.9e-01, p ≤1.e-15) (**Figure 3** and **Table 1**). In line with our previous results, this suggests that in DLBCL, key inflammasome components are induced mainly in resting (M0) and pro-inflammatory (M1) macrophages.

**Table 1:** Inflammasome components differentially expressed in the three macrophage subtypes in DLBCL.

Gene symbol	Median(Tumor)			Median(Spleen)			Fold change(Tumor/Spleen)			P value
	M0	M1	M2	M0	M1	M2	M0	M1	M2	
**AIM2**	1.039	2.520	0.701	0.019	0.001	1.724	5.5E+04	2.5E+06	4.1E-04	<1.0E-15
**ALK**	0.937	0.361	0.69	0.016	0.001	1.705	5.9E+01	3.6E+02	4.0E-04	≤5.6E-5
**CASP1**	2.703	2.23	1.818	0.137	0.001	3.194	2.0E+04	2.2E+03	5.7E-01	≤0.01
**CASP5**	0.41	1.098	0.197	0.005	0.001	0.686	8.2E+01	1.1E+06	2.9E-01	≤1.2E-13
**CARD8**	1.838	0.538	1.563	0.055	0.001	2.896	3.3E+04	5.4E+02	5.4E-01	≤4.3E-11
**CARD9**	1.199	0.468	2.179	0.024	0.001	3.598	5.0E+04	4.7E+02	6.1E-01	<1E-15
**IRF3**	1.550	0.952	1.575	0.038	0.001	2.911	4.0E+04	9.5E+02	5.4E-01	≤1.3E-3
**IRF4**	0.925	1.456	1.540	0.016	0.001	2.868	5.8E+01	1.5E+06	5.4E-01	≤2.4E-4
**IRF8**	3.015	3.865	3.217	0.185	0.001	4.697	1.6E+04	3.9E+06	6.8E-01	≤8.3E-3
NFKB1*	2.521	2.900	2.443	0.114	0.001	3.884	2.2E+04	2.9E+06	6.3E-01	<1E-15
**NFKB2**	1.009	2.222	1.232	0.018	0.001	2.485	5.6E+04	2.2E+06	5.0E-01	≤1.2E-8
**NLRP1**	0.598	0.171	0.822	0.009	0.001	1.913	6.6E+01	1.7E+02	4.3E-04	≤9E-11
**NLRP3**	0.283	0.136	1.165	0.003	0.001	2.398	9.4E+01	1.4E+02	4.9E-01	<1E-15
**NOD2**	0.652	2.242	1.881	0.01	0.001	3.266	6.5E+01	2.2E+06	5.8E-01	≤1.2E-13

Gene expression analysis [Log(TPM+1)] of the indicated inflammasome components in M0, M1 and M2 macrophages in DLBCL samples compared with spleen samples, with their tumor/spleen fold change and P value. E: exponential function. NFKB1*: no significant difference (P=0.22) in M0, M1 and M2 macrophages in DLBCL. P ≤0.05 was considered significant. To simplify the table, only the highest significant p value is shown.

### The expression levels of cytokines and TLRs are correlated with that of CD68 in DLBCL

**Figure 4:**
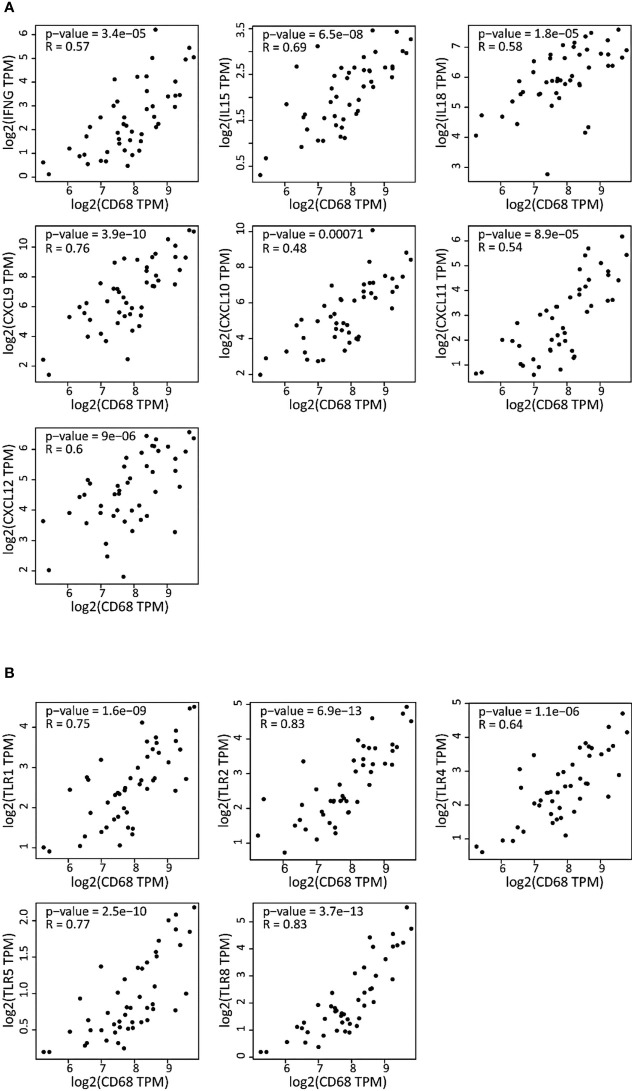
Correlation of the gene expression of cytokines and TLRs with the CD68 macrophage marker in DLBCL **(A)** Correlation between the gene expression levels of the indicated cytokines (interleukins, chemokines and chemokines receptors) and *CD68* in 47 DLBCL samples (TCGA database). **(B)** Correlation between the gene expression levels of the indicated TLRs and *CD68* in the same DLBCL samples. Quantitative comparison based on the Pearson’s correlation coefficient **(R)**. Y-axis: log2 (TPM) of the indicated gene expression level, X-axis: log2 (TPM) of *CD68* expression level. TPM, transcript count per million reads.

Then, we asked whether the high inflammatory status of the macrophage compartment in DLBCL was accompanied by increased cytokine and TLR expression in macrophages. To this aim, we investigated the correlation between the gene expression of key inflammatory cytokines (interleukins, chemokines and their receptors) and of the CD68 macrophage marker. *CD68* expression was significantly and positively correlated with the expression of pro-inflammatory cytokines (*IFNγ*, *IL15*, *IL18*, *CXCL9*, *CXCL10*, *CXCL11*, *CXCL12*) (0.48≤ R ≤0.76; p ≤0.00071) (**Figure 4A**), and TLRs (*TLR1*, *TLR2*, *TLR4*, *TLR5*, and *TLR8*) (0.64≤ R ≤0.83; p ≤1.1e-06) (**Figure 4B**). Most of these factors are involved in inflammasome priming and activation. Moreover, the Mantel-Cox survival analysis showed that expression of some cytokines and TLRs (*CCL19*, *CCR7*, *CXCR4*, *TLR2*, and *TLR5*) was associated with decreased survival (Log10 HR >0.0 and p ≤0.05), while others (*CXCL12, CXCL9, IDO1, IL18, IL6, TLR4* and *TLR8*) were associated with increased survival (Log10 HR <0.0 and p ≤0.05) of patients with DLBCL (**Figures S1C-E**). Lastly, using GSEA, we confirmed that the list of differentially expressed genes in DLBCL samples was significantly enriched in inflammation-related genes compared with control spleen samples (**Figure S1F** and **Table S2**). Overall, these results indicate a high inflammatory signature in DLBCL and a tight link between the expression of CD68 (macrophage marker) and of pro-inflammatory molecules in the DLBCL microenvironment.

### A general inflammatory state is established by M0 and M1 macrophages in DLBCL

**Figure 5:**
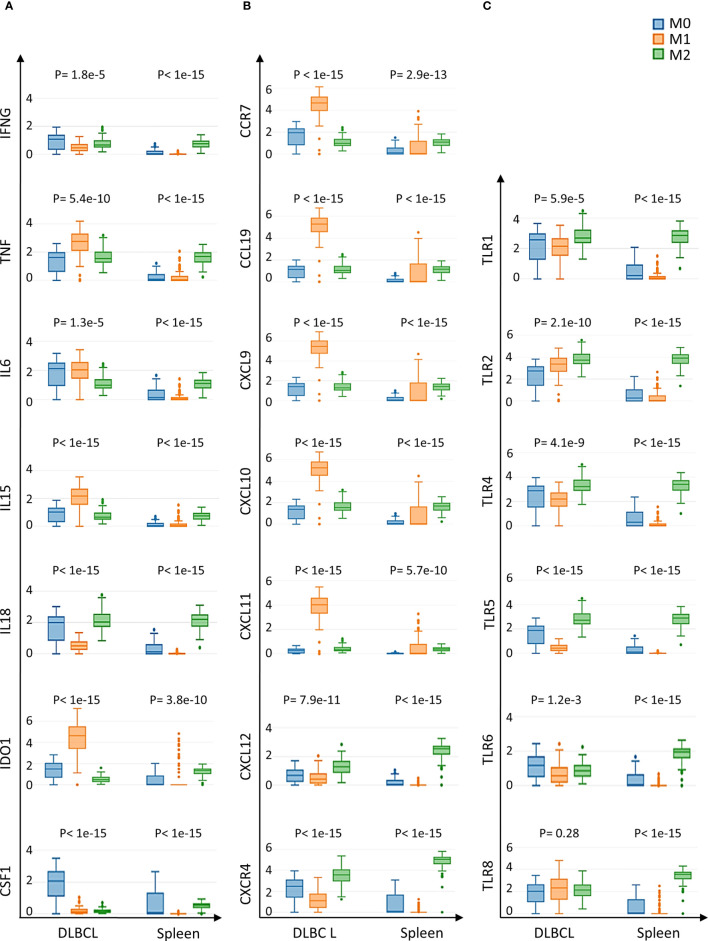
Expression of cytokines and TLRs in M0, M1 and M2 macrophages of DLBCL and spleen tissue samples **(A)** Gene expression of the indicated interleukins in M0, M1 and M2 macrophages in 47 DLBCL samples (TCGA database) and in 337 spleen tissue samples (normal secondary lymphoid organ; GTEx database). **(B)** Gene expression of the indicated chemokines and their receptors in M0, M1 and M2 macrophages in the same samples as in **(A)**. **(C)** Expression of TLR-encoding genes in M0, M1 and M2 macrophages in the same samples as in **(A)**. Y-axis: log (TPM+1) of the expression levels of the indicated gene. Additional data are in **Tables 2**, **3**. P ≤0.05 (one-way ANOVA) was considered significant. TPM, transcript count per million reads. It is important to note that the results of the deconvolution analyses in **Figures 3**, **4** display comparisons according to the tissue type (DLBCL and spleen) and not the cell type (M0, M1 and M2).

As we found a positive and significant correlation between the gene expression levels of pro-inflammatory cytokines, TLRs and CD68, we then compared their expression in M0, M1 and M2 macrophages in DLBCL and spleen (control) samples. In line with our previous results, the gene expression levels of key interleukins (*IFNγ*, *TNFα*, *IL6*, *IL15*, *IL18*, *IDO1*, and *CSF1*), chemokines and their receptors (*CCR7*, *CCL19*, *CXCL9*, *CXCL10*, *CXCL11*, *CXCL12*, *CXCR4*) (tumor/spleen fold change: 5.5e+01 to 5.1e+04 for M0; 1.0e+02 to 4.8e+06 for M1, p ≤0.01, respectively), and TLRs (*TLR1*, *TLR2*, *TLR4*, *TLR5*, *TLR6*, *TLR8*) (tumor/spleen fold change: 2.5e+04 to 5.1e+06 for M0; 2.9e+02 to 2.9e+06 for M1, p ≤1.0e-3, respectively) were significantly increased in M0 and M1 macrophages of DLBCL compared with spleen samples (**Figures 5A–C** and **Tables 2**, **3**). Conversely, their expression levels were significantly decreased in M2 macrophages of DLBCL compared with spleen samples (fold change tumor/spleen in interleukins/chemokines and their receptors: 3.3e-04 to 7.0e-01; and in TLRs: 4.3e-04 to 6.8e-01, p ≤1.0e-3). Altogether, these data demonstrated a general inflammatory state in DLBCL, specifically in the M0 and M1 macrophage compartments.

**Table 2:** Differentially expressed cytokines in the three macrophage subtypes in DLBCL.

Gene symbol	Median (Tumor)			Median (Spleen)			Fold change (Tumor/Spleen)			P value
	M0	M1	M2	M0	M1	M2	M0	M1	M2	
**CCL19**	0.765	4.840	0.662	0.012	0.001	1.660	6.4E+01	4.8E+06	4.0E-04	≤0.01
**CCR7**	1.458	4.219	0.614	0.034	0.001	1.578	4.3E+04	4.2E+06	3.9E-04	≤1.8E-3
**CSF1**	2.068	0.127	0.147	0.071	0.001	0.541	2.9E+04	1.3E+02	2.7E-01	≤2.1E-4
**CXCL9**	0.99	5.018	0.861	0.018	0.001	1.973	5.5E+01	5.0E+06	4.4E-04	≤2.1E-3
**CXCL10**	0.97	4.800	1.067	0.017	0.001	2.266	5.7E+01	4.8E+06	4.7E-01	≤1.1E-6
**CXCL11**	0.157	3.581	0.183	0.002	0.001	0.648	7.9E+01	3.6E+06	2.8E-01	≤4.1E-11
**CXCL12**	0.672	0.41	1.274	0.01	0.001	2.539	6.7E+01	4.1E+02	5.0E-01	≤7.9E-11
**CXCR4**	2.463	1.097	3.550	0.107	0.001	5.039	2.3E+04	1.1E+06	7.0E-01	≤1.0E-15
IDO1*	1.483	4.64	0.486	0.035	0.001	1.345	4.2E+04	4.6E+03	3.6E-04	≤3.8E-10
**IFNG**	0.702	0.316	0.397	0.011	0.001	1.170	6.4E+01	3.2E+02	3.4E-04	≤2.6E-4
**IL6**	1.622	1.670	0.625	0.042	0.001	1.596	3.9E+04	1.7E+06	3.9E-04	≤4.8E-8
**IL15**	0.681	1.785	0.388	0.01	0.001	1.147	6.8E+01	1.8E+06	3.4E-04	≤2.0E-15
**IL18**	1.498	0.349	1.49	0.036	0.001	2.805	4.2E+04	3.5E+02	5.3E-04	≤2.0E-13
**TNF**	1.174	2.346	1.065	0.023	0.001	2.264	5.1E+04	2.3E+06	4.7E-01	≤1.4E-9

Gene expression analysis [Log(TPM+1)] of the indicated cytokines in M0, M1 and M2 macrophages of DLBCL samples compared with spleen samples, with their tumor/spleen fold change and P value. E: Exponential function. IDO1*: no significant difference in M0, M1 and M2 macrophages (p= 0.07). P ≤0.05 was considered significant. To simplify the table, only the highest significant p value is shown.

**Table 3:** Differentially expressed TLRs in the three macrophage subtypes in DLBCL.

Gene symbol	Median (Tumor)			Median (Spleen)			Fold change (Tumor/Spleen)			P value
	M0	M1	M2	M0	M1	M2	M0	M1	M2	
TLR1*	2.035	1.763	2.089	0.068	0.001	3.499	3.0E+04	1.8E+06	6.0E-01	≤1.0E-15
**TLR2**	2.194	2.944	3.093	0.081	0.001	4.569	2.7E+04	2.9E+06	6.8E-01	≤8.7E-5
**TLR4**	2.325	1.821	2.607	0.093	0.001	4.059	2.5E+04	1.8E+06	6.4E-01	≤1.0E-3
**TLR5**	1.399	0.292	2.12	0.032	0.001	3.539	4.4E+04	2.9E+02	6.0E-04	≤1.0E-15
**TLR6**	1.173	0.574	0.85	0.023	0.001	1.956	5.1E+04	5.7E+02	4.3E-04	≤1.2E-3
TLR8*	2.014	2.320	2.130	0.067	0.001	3.544	3.0E+04	2.3E+06	6.0E-01	≤1.0E-15

Gene expression analysis [Log(TPM+1)] of TLRs in M0, M1 and M2 macrophages in DLBCL samples compared with spleen samples, with their tumor/spleen fold changes and P values. E: Exponential function. TLR1*: no significant difference in M0, M1 and M2 macrophages in DLBCL samples (p= 0.21 and p= 0.28, respectively). P ≤0.05 was considered significant. To simplify the table, only the highest significant p value is shown.

### IRF8 expression is elevated in CD68^high^ macrophages and is correlated with CD68 expression in the DLBCL microenvironment

**Figure 6:**
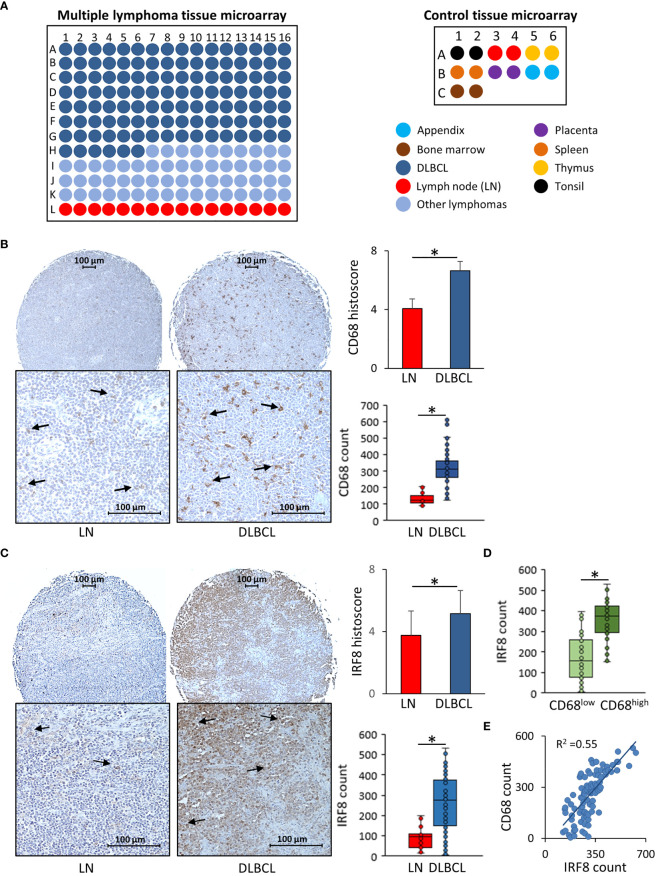
Immunohistochemical analysis of CD68 and IRF8 expression in 118 DLBCL samples and 30 normal lymphoid tissue samples **(A)** Scheme showing the distribution of tumor and normal tissue samples in each slide of the two tissue microarrays. **(B)** Representative images showing CD68 expression in normal lymph nodes (LN) and DLBCL samples (left panels). Quantitative analysis of the CD68 histoscore and absolute count in DLBCL and LN samples (right panels). **(C)** Representative images showing IRF8 expression in normal lymph nodes (LN) and DLBCL samples (left panels). Quantitative analysis of IRF8 histoscore and absolute count in DLBCL and LN samples (right panels). **(D)** IRF8 absolute count in the CD68^low^ and CD68^high^ DLBCL groups. **(E)** CD68 and IRF8 correlation analysis. *P ≤0.05.

Then, we used IHC to confirm some of our results at the protein level in a TMA that included other human DLBCL samples (**Figure 6A**). First, we evaluated CD68 expression in 118 DLBCL samples and in 30 normal lymphoid tissue samples (appendix, bone marrow, lymph nodes, placenta, spleen, thymus, and tonsil). The mean histoscore for CD68 was significantly higher in DLBCL samples than normal lymph nodes (6.63 ± 0.65 vs 4.06 ± 0.66, p= 8.5E-13) as well as the mean absolute number of CD68-positive cells (317.2 ± 99.4 vs 156.1 ± 81.9, p= 3.7E-12) (**Figure 6B**). Then, we assessed the expression of IRF8, one of the most functionally important inflammatory factors that we found upregulated in M0 and M1 macrophages in DLBCL samples (see **Figure 3**). The mean IRF8 histoscore and absolute number of positive cells were significantly higher in DLBCL than in lymph node samples (5.2 ± 1.5 vs 3.8 ± 1.6, p= 0.002; and 262.4 ± 134.7 vs 92.6 ± 55.4, p= 1.98E-12, respectively) (**Figure 6C**). Then, to investigate the association between CD68 and IRF8 expression, we first classified the samples using the median absolute number of CD68-positive cells = 310 as threshold (CD68^low^: ≤310 cells and CD68^high^: >310 cells) IRF8 absolute count was higher in the CD68^high^ group than in the CD68^low^ group (353.5 ± 89.8 vs 172.1 ± 109.5, p= 3.3E-14) (**Figure 6D**). Moreover, we found a significant and positive correlation between CD68 and IRF8 counts (R= 0.54, p= 0.002) (**Figure 6E**). Altogether, these IHC results in an independent cohort of DLBCL samples validated the higher infiltration of CD68-positive cells and the increased expression of the inflammatory factor IRF8 in tumors compared with normal lymph nodes, and also highlighted a strong correlation between these markers.

## Discussion

B-cell NHL usually develops in a specialized microenvironment characterized by the presence of different populations (including immune cells) that tightly interact with malignant B cells and that might be turned into tumor-supportive cells (19, 20). This microenvironment, in which many different cell types and factors (e.g. cytokines and chemokines) are present, influences tumor initiation, progression, and metastasis formation. It also may play a major role in the response to treatment. Recent studies indicated that immune cells are associated with DLBCL prognosis.

Here, by exploiting already available DLBCL datasets, we analyzed in an unbiased manner the infiltration score of each immune cell population in the DLBCL microenvironment and found a clear prominence of macrophages with a significantly higher proportion of M0 and M1 than M2 subpopulations in DLBCL compared with spleen controls. According to the classical M1/M2 concept, M0 macrophages can be reprogrammed into polarized M1 macrophages, a pro‐inflammatory phenotype with anti‐tumor activity, or to M2 immunosuppressive macrophages that contribute to tumor progression (17). However, the flexibly of M1/M2 polarity could completely change the outcome (21). Therefore, we hypothesized that the observed change in the cellular composition of the DLBCL microenvironment might imply a switch to a pro-inflammatory context. To test this hypothesis we performed correlation analyses using CD68, which is considered a pan-macrophage marker with a strong functional impact on tumor biology (7), and found a significant and positive correlation between CD68 expression and the expression of several inflammation factors, including IRF8. We also explored the inflammatory status of M0, M1 and M2 macrophages in DLBCL. We found that many factors (cytokines, chemokines and their receptors and TLRs) implicated in macrophage homing and in the two-step inflammasome activation model (priming and activation) were overexpressed in M0 and M1 macrophages (but not in anti-inflammatory M2 macrophages) in DLBCL samples compared with spleen controls. This again suggests an increased inflammatory response by macrophages in the DLBCL microenvironment.

We hypothesize that inflammasomes may influence macrophage polarization in DLBCL to help setting up a pro-inflammatory context necessary to rebalance the immunosuppressive tumor microenvironment usually present in lymphoma and to recover an optimal anti-tumor function. According to a study in which primary mouse macrophages were infected with *Leishmania amazonensis*, influencing the inflammasome function in macrophages affects their polarization. Specifically, these pathogens can develop molecular strategies, such as targeting histone H3 epigenetic modifications in macrophages, to modulate the NFKB/NLRP3-mediated inflammation to harness their phenotypic potential towards promoting their survival and proliferation (22). Similarly, we could hypothesize that tumor cells might modulate epigenetic modifications to turn macrophages into the most appropriate subtype for their benefit. More biological and genetic/epigenetic studies are needed to test this hypothesis in DLBCL. In addition, such epigenetic modifications might represent a rich molecular resource to understand and modulate the functionality of macrophages for anti-cancer treatment.

*NLRP1* and *3* were among the inflammasomes components with increased expression in M0 and M1 macrophages of DLBCL samples, compared with spleen. Although the type and number of inflammasomes are growing, the NLRP3 inflammasome is the prototypical and best characterized inflammasome. Yet, the precise mechanisms underlying NLRP3 activation remain debated (23, 24). For instance, some studies suggest that the anaplastic lymphoma kinase (ALK) inflammasome component is required for NLRP3 activation in macrophages (25) and that NLRP3 inflammasome activation might play a pro-tumor role through the IL-18 effector cytokine (26). In addition, some of the inflammasome components with increased expression in M0 and M1 macrophages of DLBCL samples harbor the caspase-associated recruitment domain (CARD) that is implicated in apoptosis, NFKB activation, and cytokine regulation. Specifically, CARD8 is involved in the regulation of caspase-1 and NFKB activation (27). Similarly, ALK mediates NFKB and caspase-1 activation, and NLRP3 transcription during inflammasome priming (25). As NFKB is a key regulator of the innate immune response by infiltrating immune cells, its elevated expression in M0 and M1 macrophages might modulate DLBCL immunosuppressive function. The immune system might at first trigger inflammation to counterattack the DLBCL immunosuppressive microenvironment. This might explain why the M2 cell proportion was not increased in DLBCL compared with spleen. Moreover, IL-18 is a pro-inflammatory cytokine from the IL-1 super-family that mediates inflammation in the TME, with a controversial role in the host response to tumorigenesis. For instance, this crucial NLRP3 inflammasome effector molecule could also inhibit the proliferation of cytotoxic cells (e.g. natural killer cells) to promote tumor growth and metastasis formation by inducing the expression of programmed death 1 (PD1) (26, 28). IL-15 also is an important pro-inflammatory interleukin that is produced (like IL-18) mainly by macrophages during the innate immune response and can induce the production of other pro-inflammatory molecules, such as TNFα, GM-CSF, and IFNγ (29), thus perpetuating inflammation.

Unfortunately, the survival analysis did not allow drawing any clear conclusion on whether the inflammatory genes deregulated in DLBCL samples have a pro- or anti-tumoral function. In fact, half of them had a positive association with survival, while the others had a negative association. However, this finding should be considered in the light of the significant increase in M0 and M1 macrophage subpopulations in DLBCL, suggesting that the DLBCL microenvironment may try to control the M2 immunosuppressive state.

Lastly, we validated by IHC analysis the upregulation of CD68 (pan-macrophage marker) and IRF8 (inflammation marker) in a TMA containing DLBCL samples from an independent cohort. IRF-8 is a key transcription factor that regulates macrophage differentiation and activation and that plays a tumor suppressor role. Its expression in breast cancer is strongly and positively correlated with CD8^+^ T cell infiltration, which has been associated with favorable clinical outcomes (30). Mice lacking IRF8 display defects in the induction of IL-12 and IFN-γ pro-inflammatory genes (31). In addition, IRF8 expression is a prognostic biomarker of the response to therapies, such as monoclonal antibodies (30).

Altogether, our *in silico* analysis indicates that key pro-inflammatory molecules, such as inflammasome components, cytokines and TLRs, are overexpressed in M0 (that can be polarized to M1 cells) and M1 macrophages, the most abundant macrophage subpopulations in the DLBCL microenvironment, thus creating a high inflammatory state. Our results set the basis for a more in-depth study of all these inflammatory markers and their relationship with the phenotypes of macrophages that infiltrate the DLBCL microenvironment. On the basis of our results, we propose a model of the molecular events that might occur in DLBCL (**Figure S2**).

The role of inflammasomes in cancer and specifically in DLBCL is not completely understood and sometimes results are controversial. The mechanisms and signaling events that mediate its priming, activation and assembly remain uncertain. Therefore, it is important to thoroughly investigate the inflammasome role in DLBCL pathogenesis and its impact on macrophage differentiation. As inflammasomes have a role in cancer, specific nanotargeting or modulation of inflammasome components in macrophages might open new avenues for DLBCL treatment, while reducing drug toxicity (32, 33).

## Data availability statement

The datasets presented in this study can be found in online repositories. The names of the repository/repositories and accession number(s) can be found in the article/**Supplementary Material**.

## Author contributions

MSB conceived the original idea, generated the bioinformatics data, wrote, corrected and finalized the manuscript. LS generated the figures. PA and MC participated in the generation of complementary bioinformatics data, gave critical feedback and revised the manuscript. MB brought bioinformatics skills and critical feedback. All authors contributed to the article and approved the submitted version.

## Supplementary material

Supplementary Figure 1
**(A)** Proportion of CD4 T cells, regulatory T cells (Tregs), neutrophils (Neuφ), CD8 T cells, dendritic cells (DC), and M0, M1 and M2 macrophages in 47 DLBCL samples (TCGA database) and 337 spleen tissue samples (GTEx database). The proportions of the different immune cell subtypes in DLBCL and spleen samples were compared with one-way ANOVA. **(B)** Proportion of M0, M1 and M2 macrophages in 47 DLBCL samples (TCGA database) and 337 spleen tissue samples (GTEx database). One-way ANOVA was used to compare the proportion of M0, M1 and M2 in DLBCL and spleen. **(C)** Survival significance of inflammasome components in DLBCL. **(D)** Survival significance in DLBCL of key cytokines involved in inflammation (interleukins, chemokines and chemokine receptors). **(E)** Survival significance in DLBCL of key TLRs. Results are displayed as log10 hazard ratio (HR), estimated using the Mantel–Cox test and p ≤0.05 was considered significant. **(F)** Gene set enrichment analysis showing the enrichment of inflammatory genes in transcriptomic data of DLBCL samples compared with control spleen. FDR: false discovery rate.

Supplementary Figure 2 Proposed model of the molecular events underlying M1-related activation of the inflammasome in DLBCL Compared with spleen (normal secondary lymphoid organ), in DLBCL the proportion of M1 macrophages is increased and most of the inflammasome canonical pathway components are upregulated in M1 macrophages. Inflammasome priming and activation are required for their assembly and activity. The priming step can be seen as a necessary regulation to avoid unwanted activation. It allows the transcriptional upregulation of proteins required for inflammasome assembly and downstream signaling. In our hypothetic model, stimulation of TNFα and IFNγ receptors (R) and TLRs triggers in the nucleus the NFκB-mediated transcriptional upregulation of NLRP1, 3 and caspase-1, 4 and 5, and the IRF3,4 and 8-mediated transcriptional upregulation of NAIP and BGP1, 3, 4. Then, the activation step induces post-translational modifications of the primed sensors (NLRP1 and 3, NLRC4, AIM2, NOD2 and TRIM20), resulting in conformational changes that are the starting point for the assembly of platforms that are unique to each inflammasome with specific adaptors (e.g. ALK, CARD8 and 9). In our model, the canonical pathway is predominant. Once assembled, the inflammasome complex promotes the inflammatory response that can play an anti-tumor role in DLBCL, leading to increased gene expression of pro-inflammatory cytokines, such as interleukins (*IL18, IL15, IL6, IDO1, CSF1*), chemokines and their receptors (*CXCL9, CXL10, CXCL11, CXCL12, CXCR4, CCL19*, and *CCR7*). More studies are needed to determine whether the induced inflammatory response contributes to limit or promote cancer progression.

Supplementary Table 1 List of the characteristics of patients with DLBCL from the TCGA database.

Supplementary Table 2 Manually curated list of the inflammation-related genes used in this analysis in DLBCL samples versus spleen samples with their respective fold change and P values. They were used for the GSEA analysis in **Figure S1F**. P ≤0.05 was considered significant.

Supplementary Table 3 List of the characteristics of the patients whose tumor samples were included in the tissue microarray used for the immunohistochemistry analysis.

Supplementary Table 4 Proportion of M0, M1 and M2 macrophages **(A)**, regulatory T cells (Treg), CD4 T cells and CD8 T cells **(B)**, dendritic cells (DC), natural killer cells (NK) and neutrophils (Neuφ) **(C)** in 47 DLBCL samples and 337 spleens (normal secondary lymphoid organ) tissue samples, with their fold change and P value, assessed using the GEPIA2 deconvolution tool. E: Exponential function. P ≤0.05 was considered significant.

## Glossary

**Table:**

ALK	anaplastic lymphoma kinase
B	B cells
CASP1	caspase 1
CARD8 and 9	caspase-associated recruitment domain 8 and 9
CCL	C-C motif chemokine ligand
CD4T	CD4 T cells
CD8T	CD8 T cells
CCR	C-C chemokine receptor
CSF1	macrophages colony stimulating factor 1
CXCL	C-X-C motif chemokine ligand
DC	dendritic cells
DLBCL	diffuse large B cell lymphoma
GBP	guanylate binding protein
GM-CSF	granulocyte macrophages colony stimulating factor
IDO1	Indoleamine-pyrrole 2,3-dioxygenase 1
IL	interleukin
4 and 8	Interferon regulatory factor 3
iTreg	induced regulatory T cells
MO	monocytes
MФ	macrophages
NAIP	NLR family apoptosis inhibitory protein
Neuφ	neutrophils
NFKB	nuclear factor kappa-light-chain-enhancer of activated B cells
NHL	non-hodgkin lymphoma
NK	natural killer cells
NKT	natural killer T cells
NLRC4	NLR family CARD domain 4
NOD2	nucleotide-binding oligomerization domain 2
nTreg	natural regulatory T cells
TAMs	tumor associated macrophages
TLR	toll like receptor
Tfh	T follicular helper cells
Th1	T helper 1 cells
Th2	T helper 2 cells
Th17	T helper 17 cells
TME	tumor microenvironment
TRIM20	tripartite motif family.
