# Supplementary figures and images for: Diffuse large B-cell lymphoma microenvironment displays a predominant macrophage infiltrate marked by a strong inflammatory signature
Source: Front Immunol. 2023 May 2;14:1048567. doi: 10.3389/fimmu.2023.1048567 (PMC10185825; doi:10.3389/fimmu.2023.1048567)

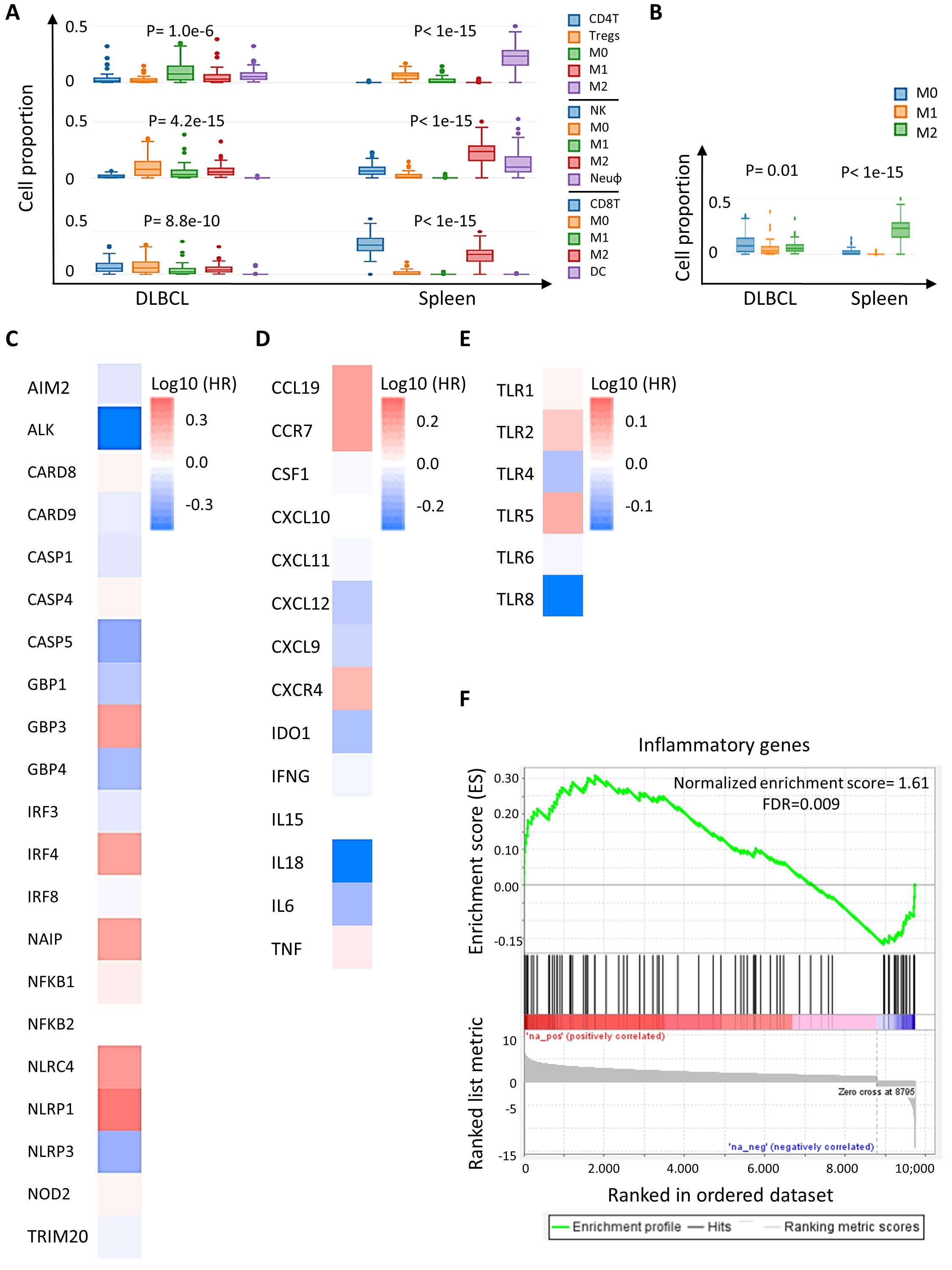

Supplement: Supplementary Figure 1 — (A) Proportion of CD4 T cells, regulatory T cells (Tregs), neutrophils (Neuφ), CD8 T cells, dendritic cells (DC), and M0, M1 and M2 macrophages in 47 DLBCL samples (TCGA database) and 337 spleen tissue samples (GTEx database). The proportions of the different immune cell subtypes in DLBCL and spleen samples were compared with one-way ANOVA. (B) Proportion of M0, M1 and M2 macrophages in 47 DLBCL samples (TCGA database) and 337 spleen tissue samples (GTEx database). One-way ANOVA was used to compare the proportion of M0, M1 and M2 in DLBCL and spleen. (C) Survival significance of inflammasome components in DLBCL. (D) Survival significance in DLBCL of key cytokines involved in inflammation (interleukins, chemokines and chemokine receptors). (E) Survival significance in DLBCL of key TLRs. Results are displayed as log10 hazard ratio (HR), estimated using the Mantel–Cox test and p ≤0.05 was considered significant. (F) Gene set enrichment analysis showing the enrichment of inflammatory genes in transcriptomic data of DLBCL samples compared with control spleen. FDR: false discovery rate. [file Image_1.tif]

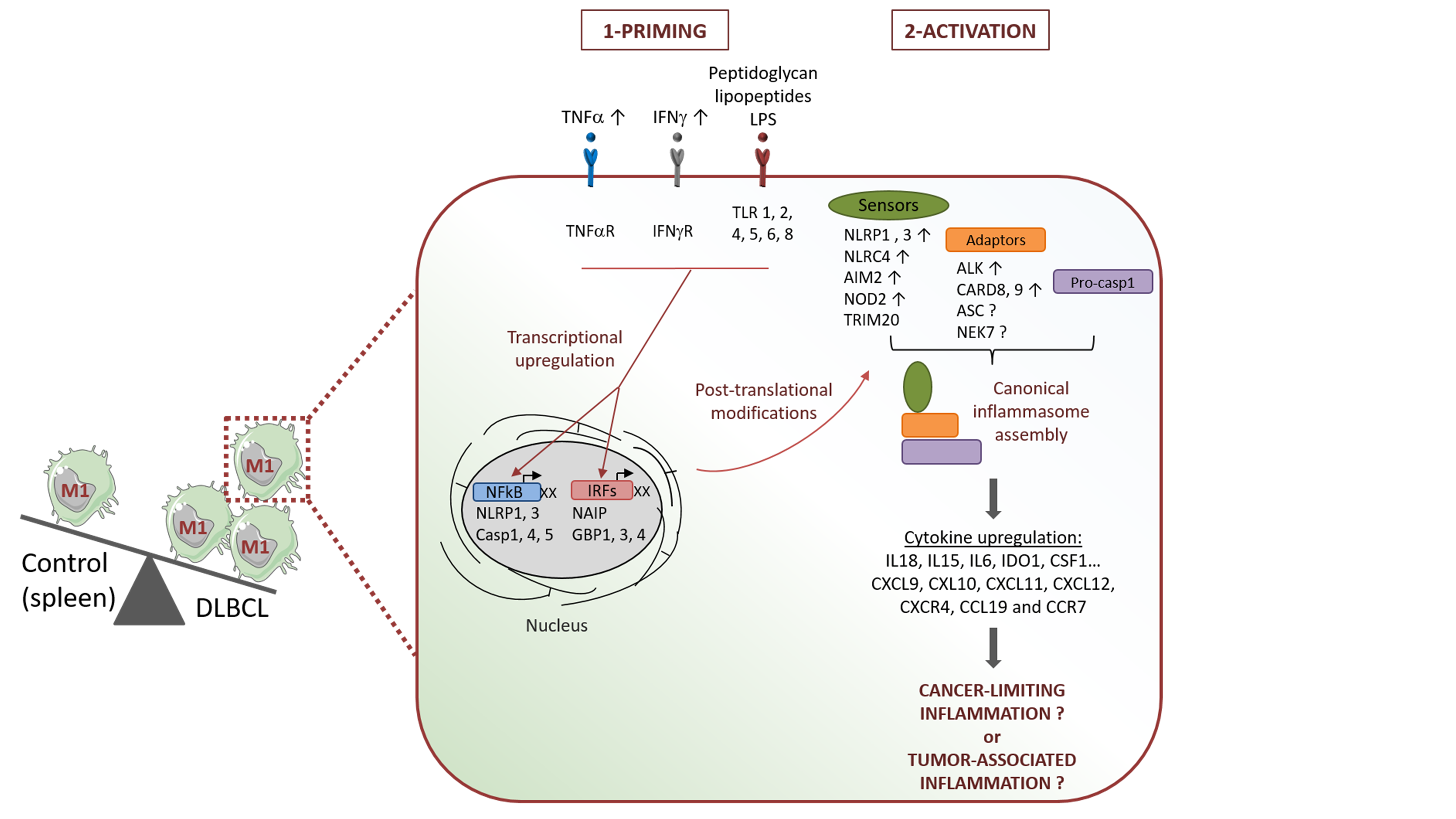

Supplement: Supplementary Figure 2 — Proposed model of the molecular events underlying M1-related activation of the inflammasome in DLBCL Compared with spleen (normal secondary lymphoid organ), in DLBCL the proportion of M1 macrophages is increased and most of the inflammasome canonical pathway components are upregulated in M1 macrophages. Inflammasome priming and activation are required for their assembly and activity. The priming step can be seen as a necessary regulation to avoid unwanted activation. It allows the transcriptional upregulation of proteins required for inflammasome assembly and downstream signaling. In our hypothetic model, stimulation of TNFα and IFNγ receptors (R) and TLRs triggers in the nucleus the NFκB-mediated transcriptional upregulation of NLRP1, 3 and caspase-1, 4 and 5, and the IRF3,4 and 8-mediated transcriptional upregulation of NAIP and BGP1, 3, 4. Then, the activation step induces post-translational modifications of the primed sensors (NLRP1 and 3, NLRC4, AIM2, NOD2 and TRIM20), resulting in conformational changes that are the starting point for the assembly of platforms that are unique to each inflammasome with specific adaptors (e.g. ALK, CARD8 and 9). In our model, the canonical pathway is predominant. Once assembled, the inflammasome complex promotes the inflammatory response that can play an anti-tumor role in DLBCL, leading to increased gene expression of pro-inflammatory cytokines, such as interleukins (IL18, IL15, IL6, IDO1, CSF1), chemokines and their receptors (CXCL9, CXL10, CXCL11, CXCL12, CXCR4, CCL19, and CCR7). More studies are needed to determine whether the induced inflammatory response contributes to limit or promote cancer progression. [file Image_2.tif]
