# Supplementary material for: Diffuse large B-cell lymphoma microenvironment displays a predominant macrophage infiltrate marked by a strong inflammatory signature
Source: Front Immunol. 2023 May 2;14:1048567. doi: 10.3389/fimmu.2023.1048567 (PMC10185825; doi:10.3389/fimmu.2023.1048567)
Supplement: Supplementary Table 2 — Manually curated list of the inflammation-related genes used in this analysis in DLBCL samples versus spleen samples with their respective fold change and P values. They were used for the GSEA analysis in Figure S1F . P ≤0.05 was considered significant. [file DataSheet_2.pdf]

Table S2.

Gene Symbol	Gene ID	Median (Tumor)	Median (Normal)	Log2(Fold Change)	Adj p
AIM2	ENSG00000163568.13	51.499	3.480	3.551	2.96e-22
ARG1	ENSG00000118520.13	0.020	64.359	-6.002	1.45e-52
ASCC3	ENSG00000112249.13	8.400	1.000	2.233	4.03e-49
BCL2	ENSG00000171791.11	8.400	0.950	2.269	5.14e-28
BCL6	ENSG00000113916.17	29.790	164.426	-2.426	1.64e-14
CARD11	ENSG00000198286.9	46.179	5.610	2.835	3.10e-29
CASP3	ENSG00000164305.17	24.501	3.440	2.522	3.51e-30
CASP5	ENSG00000137757.10	0.420	2.970	-1.483	3.21e-10
CASP6	ENSG00000138794.9	7.860	1.570	1.786	1.21e-20
CASP7	ENSG00000165806.19	10.030	1.370	2.218	6.35e-34
CASP9	ENSG00000132906.17	10.890	4.160	1.204	1.64e-14
CCL14	ENSG00000276409.4	7.520	0.520	2.487	4.20e-26
CCL17	ENSG00000102970.10	7.800	0.000	3.138	1.09e-49
CCL18	ENSG00000275385.1	160.863	0.080	7.228	9.26e-119
CCL19	ENSG00000172724.11	242.089	0.050	7.855	2.97e-131
CCL2	ENSG00000108691.9	50.380	0.200	5.420	1.76e-89
CCL21	ENSG00000137077.7	63.831	0.110	5.868	1.39e-69

CCL22	ENSG00000102962.4	9.220	0.020	3.325	9.67e-72
CCL3	ENSG00000277632.1	39.689	13.900	1.449	3.29e-11
CCL4	ENSG00000275302.1	45.981	17.950	1.310	1.20e-6
CCL8	ENSG00000108700.4	2.040	0.000	1.604	5.20e-53
CCR10	ENSG00000184451.5	2.380	0.180	1.518	3.60e-40
CCR2	ENSG00000121807.5	1.820	6.830	-1.473	1.70e-7
CCR5	ENSG00000160791.13	8.500	0.830	2.376	4.91e-30
CCR7	ENSG00000126353.3	29.181	8.670	1.642	2.18e-4
CD34	ENSG00000174059.16	8.210	0.210	2.928	1.11e-77
CD38	ENSG00000004468.12	20.141	1.370	3.157	5.50e-45
CSF1	ENSG00000184371.13	9.960	1.160	2.343	1.03e-38
CXCL10	ENSG00000169245.5	34.489	0.060	5.065	7.73e-107
CXCL11	ENSG00000169248.12	6.100	0.000	2.828	2.64e-81
CXCL12	ENSG00000107562.16	23.169	0.210	4.320	7.77e-102
CXCL13	ENSG00000156234.7	34.071	0.010	5.118	1.34e-120
CXCL14	ENSG00000145824.12	4.920	0.140	2.377	1.37e-50
CXCL9	ENSG00000138755.5	117.002	0.030	6.840	3.66e-131
CXCR1	ENSG00000163464.7	0.030	243.824	-7.893	1.71e-90
CXCR2	ENSG00000180871.7	0.050	117.083	-6.813	3.35e-69
CXCR3	ENSG00000186810.7	14.760	1.090	2.915	7.58e-33
CXCR6	ENSG00000172215.5	13.580	0.890	2.948	2.90e-33

GBP1	ENSG00000117228.9	35.641	4.610	2.707	4.32e-20
GBP3	ENSG00000117226.11	6.080	1.380	1.573	1.24e-15
GBP4	ENSG00000162654.8	30.049	1.860	3.440	4.17e-39
GSDMD	ENSG00000104518.10	136.598	44.129	1.608	6.97e-19
HMGB1	ENSG00000189403.14	413.974	37.530	3.429	4.80e-43
HMGB1P5	ENSG00000132967.9	85.727	7.350	3.377	6.24e-41
IDO1	ENSG00000131203.12	26.270	0.220	4.482	1.22e-69
IFNG	ENSG00000111537.4	3.300	0.210	1.829	1.54e-9
IL10	ENSG00000136634.5	4.830	1.110	1.466	2.22e-14
IL15	ENSG00000164136.16	3.580	0.770	1.372	1.82e-14
IL18	ENSG00000150782.11	56.590	3.670	3.624	2.08e-62
IL1B	ENSG00000125538.11	2.350	10.970	-1.837	1.85e-13
IL6	ENSG00000136244.11	1.660	0.110	1.261	3.00e-23
IRF3	ENSG00000126456.15	119.460	30.651	1.928	1.66e-35
IRF4	ENSG00000137265.14	21.640	0.820	3.637	1.98e-58
IRF8	ENSG00000140968.10	116.994	9.410	3.503	7.25e-48
LDHA	ENSG00000134333.13	583.989	178.131	1.707	7.79e-14
LDHB	ENSG00000111716.12	659.394	38.521	4.063	2.99e-55
LRR1	ENSG00000165501.16	19.830	1.290	3.185	4.24e-78
NAIP	ENSG00000249437.7	5.130	47.110	-2.972	2.16e-17
NFKB1	ENSG00000109320.11	29.850	9.190	1.598	1.43e-9

NFKB2	ENSG00000077150.17	67.032	20.440	1.666	5.77e-17
NLRC4	ENSG00000091106.18	1.760	10.070	-2.004	1.43e-14
NLRP1	ENSG00000091592.15	11.880	29.219	-1.230	1.72e-9
NLRP3	ENSG00000162711.16	0.850	11.820	-2.793	2.98e-23
NLRP6	ENSG00000174885.12	0.210	5.990	-2.530	1.04e-25
NOD2	ENSG00000167207.11	2.090	6.500	-1.279	2.35e-7
PRR11	ENSG00000068489.12	11.830	0.630	2.977	9.66e-70
PRR12	ENSG00000126464.13	6.660	1.390	1.680	2.57e-27
PRR22	ENSG00000212123.3	2.150	0.350	1.222	9.62e-38
PRR3	ENSG00000204576.11	12.320	1.920	2.189	2.11e-46
PRR33	ENSG00000184682.5	1.620	8.880	-1.915	1.76e-17
TGFA	ENSG00000163235.15	0.520	2.570	-1.232	1.75e-12
TGFB3	ENSG00000119699.7	4.600	0.330	2.074	1.67e-62
TGFBI	ENSG00000120708.16	61.201	21.240	1.484	4.15e-10
TLR1	ENSG00000174125.7	4.470	17.540	-1.761	2.06e-10
TLR10	ENSG00000174123.10	13.490	1.440	2.570	3.06e-17
TLR2	ENSG00000137462.6	3.980	66.758	-3.766	7.70e-28
TLR4	ENSG00000136869.13	4.530	14.420	-1.479	2.39e-9
TLR5	ENSG00000187554.11	0.700	8.440	-2.473	4.48e-18
TLR8	ENSG00000101916.11	2.170	14.620	-2.301	2.00e-10
TNF	ENSG00000232810.3	6.380	1.440	1.597	2.79e-15

Table S4.

A

	Median (Tumor)			Median (Spleen)			Fold change (Tumor/Spleen)			P value
	M0	M1	M2	M0	M1	M2	M0	M1	M2	
**Cell proportion**	0.075	0.029	0.051	0.008	0.001	0.233	9.4E+01	2.9E+01	2.2E-01	≤1.7E-5

B

	Median (Tumor)			Median (Spleen)			Fold change (Tumor/Spleen)			P value
	Treg	CD4T	CD8T	Treg	CD4T	CD8T	Treg	CD4T	CD8T	
**Cell proportion**	0.015	0.013	0.071	0.062	0.001	0.341	2.4E-01	1.3E+01	2.1E-01	≤1.0E-6

C

	Median (Tumor)			Median (Spleen)			Fold change (Tumor/Spleen)			P value
	DC	NK	Neuφ	DC	NK	Neuφ	DC	NK	Neuφ	
**Cell proportion**	0.001	0.007	0.001	0.001	0.061	0.095	1.0E+00	1.1E-01	1.1E-02	≤1.0E-6
