# Supplementary material for: Diffuse large B-cell lymphoma microenvironment displays a predominant macrophage infiltrate marked by a strong inflammatory signature
Source: Front Immunol. 2023 May 2;14:1048567. doi: 10.3389/fimmu.2023.1048567 (PMC10185825; doi:10.3389/fimmu.2023.1048567)
Supplement: Supplementary Table 3 — List of the characteristics of the patients whose tumor samples were included in the tissue microarray used for the immunohistochemistry analysis. [file DataSheet_3.pdf]

Pos.	No.	Age	Sex	Organ/Anatomic Site	Pathology diagnosis	Type
A1	1	63	F	Lymph node	Diffuse small B-cell lymphoma of right armpit	Malignant
A2	2	77	M	Lymph node	Diffuse small B-cell lymphoma of right armpit	Malignant
A3	3	68	M	Lymph node	Diffuse large B-cell lymphoma of left groin	Malignant
A4	4	15	M	Lymph node	Diffuse B-cell lymphoma of right elbow joint	Malignant
A5	5	51	M	Lymph node	Diffuse small B-cell lymphoma of right groin	Malignant
A6	6	28	M	Lymph node	Diffuse large B-cell lymphoma	Malignant
A7	7	57	F	Lymph node	Diffuse B-cell lymphoma of right neck	Malignant
A8	8	29	F	Lymph node	Diffuse B-cell lymphoma of left armpit	Malignant
A9	9	40	M	Lymph node	Diffuse small B non-cleaved cell lymphoma of mediastinum	Malignant
A10	10	41	F	Lymph node	Diffuse B-cell lymphoma of neck	Malignant
A11	11	43	M	Lymph node	Diffuse B-cell lymphoma of mesentery	Malignant
A12	12	50	M	Lymph node	Diffuse small B-cell lymphoma of retroperitoneum	Malignant
A13	13	31	M	Lymph node	Diffuse large B-cell lymphoma of left leg	Malignant
A14	14	50	F	Lymph node	Diffuse large B-cell lymphoma of stomach	Malignant
A15	15	35	F	Lymph node	Diffuse B-cell lymphoma of retroperitoneum	Malignant
A16	16	50	F	Lymph node	Diffuse B-cell lymphoma of neck	Malignant
B1	17	45	M	Lymph node	Diffuse B-cell lymphoma of left groin	Malignant
B2	18	13	M	Lymph node	Diffuse B-cell lymphoma of right lower abdominal part	Malignant
B3	19	45	M	Lymph node	Diffuse large B-cell lymphoma of left groin	Malignant
B4	20	64	F	Lymph node	Diffuse large B-cell lymphoma of right armpit	Malignant
B5	21	52	M	Lymph node	Diffuse large B-cell lymphoma of right groin	Malignant
B6	22	62	F	Lymph node	Diffuse B-cell lymphoma of left armpit	Malignant
B7	23	75	F	Lymph node	Diffuse B-cell lymphoma of left armpit	Malignant
B8	24	50	F	Lymph node	Diffuse B-cell lymphoma of left palate	Malignant
B9	25	59	M	Lymph node	Diffuse B-cell lymphoma of left thigh	Malignant
B10	26	78	F	Lymph node	Diffuse small B-cell lymphoma of left upper eyelid	Malignant
B11	27	30	M	Lymph node	Diffuse B-cell lymphoma of left neck	Malignant
B12	28	10	M	Lymph node	Diffuse B-cell lymphoma of left neck	Malignant
B13	29	76	F	Lymph node	Diffuse B-cell lymphoma of post aurem	Malignant
B14	30	76	M	Lymph node	Diffuse large B-cell lymphoma of left abdominal part	Malignant
B15	31	52	M	Lymph node	Diffuse B-cell lymphoma over clavicle	Malignant
B16	32	36	F	Lymph node	Diffuse B-cell lymphoma of right breast	Malignant
C1	33	64	F	Lymph node	Diffuse B-cell lymphoma of left groin	Malignant
C2	34	62	M	Lymph node	Diffuse B-cell lymphoma of left neck	Malignant
C3	35	68	F	Lymph node	Diffuse B-cell lymphoma of right groin	Malignant

C4	36	85	M	Lymph node	Diffuse B-cell lymphoma	Malignant
C5	37	51	F	Lymph node	Diffuse B-cell lymphoma of left thyroid gland	Malignant
C6	38	52	F	Lymph node	Diffuse large B-cell lymphoma of right armpit	Malignant
C7	39	53	M	Lymph node	Diffuse B-cell lymphoma of spleen	Malignant
C8	40	71	M	Lymph node	Diffuse large B-cell lymphoma of right neck	Malignant
C9	41	8	M	Lymph node	Diffuse B-cell lymphoma of left armpit	Malignant
C10	42	50	M	Lymph node	Diffuse B-cell lymphoma of left armpit	Malignant
C11	43	51	M	Lymph node	Diffuse B-cell lymphoma of mediastinum	Malignant
C12	44	50	M	Lymph node	Diffuse large B-cell lymphoma of left groin	Malignant
C13	45	61	F	Lymph node	Diffuse B-cell lymphoma of right groin	Malignant
C14	46	40	M	Lymph node	Diffuse B-cell lymphoma of right armpit	Malignant
C15	47	24	M	Lymph node	Diffuse B-cell lymphoma of neck	Malignant
C16	48	48	F	Lymph node	Diffuse B-cell lymphoma of left groin	Malignant
D1	49	52	M	Lymph node	Diffuse B-cell lymphoma of groin	Malignant
D2	50	51	F	Lymph node	Diffuse small B-cell lymphoma of submaxilla	Malignant
D3	51	51	M	Lymph node	Diffuse B-cell lymphoma of right armpit	Malignant
D4	52	71	M	Lymph node	Diffuse B-cell lymphoma of neck	Malignant
D5	53	42	M	Lymph node	Diffuse B-cell lymphoma of left groin	Malignant
D6	54	41	F	Lymph node	Diffuse large B-cell lymphoma of left armpit	Malignant
D7	55	38	M	Lymph node	Diffuse B-cell lymphoma of groin	Malignant
D8	56	38	M	Lymph node	Diffuse large B-cell lymphoma over clavicle	Malignant
D9	57	36	M	Lymph node	Diffuse B-cell lymphoma of right armpit	Malignant
D10	58	43	F	Lymph node	Diffuse B-cell lymphoma of left groin	Malignant
D11	59	65	M	Lymph node	Diffuse B-cell lymphoma of armpit	Malignant
D12	60	36	M	Lymph node	Diffuse B-cell lymphoma of left groin	Malignant
D13	61	56	M	Lymph node	Diffuse large B-cell lymphoma	Malignant
D14	62	38	M	Lymph node	Diffuse large B-cell lymphoma of neck	Malignant
D15	63	53	M	Lymph node	Diffuse B-cell lymphoma of neck	Malignant
D16	64	30	M	Lymph node	Diffuse B-cell lymphoma of left neck	Malignant
E1	65	40	M	Lymph node	Diffuse B-cell lymphoma of left groin	Malignant
E2	66	30	M	Lymph node	Diffuse B-cell lymphoma of left armpit	Malignant
E3	67	48	F	Lymph node	Diffuse large B-cell lymphoma of left groin	Malignant
E4	68	44	F	Lymph node	Diffuse B-cell lymphoma of left groin	Malignant
E5	69	40	M	Lymph node	Diffuse B-cell lymphoma of left neck	Malignant
E6	70	39	M	Lymph node	Diffuse B-cell lymphoma of right groin	Malignant
E7	71	36	M	Lymph node	Diffuse large B-cell lymphoma of left groin	Malignant
E8	72	42	F	Lymph node	Diffuse B-cell lymphoma of right lower limb	Malignant
E9	73	36	F	Lymph node	Diffuse B-cell lymphoma of left groin	Malignant
E10	74	57	M	Lymph node	Diffuse B-cell lymphoma of right groin	Malignant
E11	75	67	M	Lymph node	Diffuse small B-cell lymphoma	Malignant

E12	76	62	M	Lymph node	Diffuse large B-cell lymphoma of right groin	Malignant
E13	77	3	M	Lymph node	B lymphocytic lymphoma of left neck	Malignant
E14	78	33	F	Lymph node	Diffuse B-cell lymphoma of right neck	Malignant
E15	79	47	F	Lymph node	Diffuse B-cell lymphoma right submaxilla	Malignant
E16	80	67	M	Lymph node	Diffuse small B-cell lymphoma of right armpit	Malignant
F1	81	47	M	Lymph node	Diffuse large B-cell lymphoma of right neck	Malignant
F2	82	47	M	Lymph node	Diffuse B-cell lymphoma of right neck	Malignant
F3	83	68	M	Lymph node	Diffuse B-cell lymphoma of right groin	Malignant
F4	84	58	F	Lymph node	Diffuse B-cell lymphoma over right clavicle	Malignant
F5	85	32	M	Lymph node	Diffuse B-cell lymphoma of left groin	Malignant
F6	86	44	M	Lymph node	Diffuse B-cell lymphoma of right groin	Malignant
F7	87	41	M	Lymph node	Diffuse large B-cell lymphoma of left neck	Malignant
F8	88	39	M	Lymph node	Diffuse large B-cell lymphoma of right groin	Malignant
F9	89	48	F	Lymph node	Diffuse B-cell lymphoma of right groin	Malignant
F10	90	71	M	Lymph node	Diffuse B-cell lymphoma of abdominal part	Malignant
F11	91	27	M	Lymph node	Diffuse B-cell lymphoma of neck	Malignant
F12	92	51	M	Lymph node	Diffuse B-cell lymphoma of right neck	Malignant
F13	93	54	F	Lymph node	Diffuse B-cell lymphoma of armpit	Malignant
F14	94	40	F	Lymph node	Diffuse B-cell lymphoma of right lower limb	Malignant
F15	95	48	F	Lymph node	Diffuse large B-cell lymphoma of right armpit	Malignant
F16	96	45	F	Lymph node	Diffuse B-cell lymphoma of left forehead	Malignant
G1	97	69	F	Lymph node	Diffuse large B-cell lymphoma of right parotid gland	Malignant
G2	98	33	M	Lymph node	Diffuse large B-cell lymphoma over left clavicle	Malignant
G3	99	40	F	Lymph node	Diffuse large B-cell lymphoma of left groin	Malignant
G4	100	52	F	Lymph node	Diffuse large B-cell lymphoma of left neck	Malignant
G5	101	63	M	Lymph node	Diffuse B-cell lymphoma of left armpit	Malignant
G6	102	50	F	Lymph node	Diffuse B-cell lymphoma	Malignant
G7	103	72	M	Lymph node	Diffuse B-cell lymphoma of groin	Malignant
G8	104	39	M	Lymph node	Diffuse B-cell lymphoma of right armpit	Malignant
G9	105	65	M	Lymph node	Diffuse large B-cell lymphoma of groin	Malignant
G10	106	56	M	Lymph node	Nodular B-cell lymphoma of left submaxilla	Malignant
G11	107	65	F	Lymph node	Diffuse B-cell lymphoma of neck	Malignant
G12	108	74	M	Lymph node	Diffuse B-cell lymphoma of right popliteal fossa	Malignant
G13	109	54	M	Lymph node	Diffuse B-cell lymphoma of left armpit	Malignant
G14	110	62	M	Lymph node	Diffuse B-cell lymphoma of left groin	Malignant
G15	111	3	M	Lymph node	Diffuse B-cell lymphoma of retroperitoneum	Malignant

G16	112	48	M	Lymph node	Diffuse B-cell lymphoma	Malignant
H1	113	80	M	Lymph node	Diffuse B-cell lymphoma of neck	Malignant
H2	114	67	M	Lymph node	Diffuse B-cell lymphoma of right armpit	Malignant
H3	115	36	F	Lymph node	Diffuse large B-cell lymphoma of groin	Malignant
H4	116	38	F	Lymph node	Diffuse large B-cell lymphoma of retroperitoneum	Malignant
H5	117	69	M	Lymph node	Diffuse small B-cell lymphoma of right armpit	Malignant
H6	118	43	F	Lymph node	Diffuse small B-cell lymphoma of retroperitoneum	Malignant
H7	119	50	F	Lymph node	Burkitt-like lymphoma of pelvic cavity	Malignant
H8	120	71	M	Lymph node	Burkitt-like lymphoma of submaxilla	Malignant
H9	121	38	F	Lymph node	Burkitt-like lymphoma of left pelvic cavity	Malignant
H10	122	21	M	Lymph node	Follicular lymphoma of left armpit	Malignant
H11	123	38	F	Lymph node	Follicular lymphoma of right armpit	Malignant
H12	124	60	M	Lymph node	Follicular lymphoma of groin	Malignant
H13	125	50	F	Lymph node	Follicular lymphoma of right neck	Malignant
H14	126	68	F	Lymph node	Follicular lymphoma of left groin	Malignant
H15	127	62	F	Lymph node	Mantle cell lymphoma of left armpit	Malignant
H16	128	50	M	Lymph node	Plasma cell lymphoma of right groin	Malignant
I1	129	63	F	Lymph node	Plasma cell lymphoma of right abdominal part	Malignant
I2	130	70	M	Lymph node	Plasma cell lymphoma of neck	Malignant
I3	131	50	M	Lymph node	Plasma cell lymphoma of chest wall	Malignant
I4	132	45	M	Lymph node	Anaplastic large cell lymphoma of armpit	Malignant
I5	133	40	F	Lymph node	Anaplastic large cell lymphoma of right groin	Malignant
I6	134	76	F	Lymph node	Anaplastic large cell lymphoma of left neck	Malignant
I7	135	64	M	Lymph node	Anaplastic large cell lymphoma of right groin	Malignant
I8	136	26	M	Lymph node	Anaplastic large cell lymphoma of neck	Malignant
I9	137	42	M	Lymph node	Anaplastic large cell lymphoma of abdominal cavity	Malignant
I10	138	23	F	Lymph node	Anaplastic large cell lymphoma of left groin	Malignant
I11	139	75	M	Lymph node	T-cell lymphoma of right armpit	Malignant
I12	140	72	M	Lymph node	T-cell lymphoma of left neck	Malignant
I13	141	40	F	Lymph node	T-cell lymphoma of right armpit	Malignant
I14	142	41	M	Lymph node	T-cell lymphoma of groin	Malignant
I15	143	68	M	Lymph node	T-cell lymphoma	Malignant
I16	144	55	M	Lymph node	T-cell lymphoma of right armpit	Malignant
J1	145	38	M	Lymph node	T-cell lymphoma left groin	Malignant
J2	146	63	F	Lymph node	T-cell lymphoma of right lower abdominal part	Malignant
J3	147	52	M	Lymph node	T-cell lymphoma of left neck	Malignant
J4	148	44	F	Lymph node	T-cell lymphoma of left neck	Malignant
J5	149	52	M	Lymph node	Nodular T-cell lymphoma over right clavicle	Malignant
J6	150	40	F	Lymph node	T-cell lymphoma of right lower limb	Malignant
J7	151	76	M	Lymph node	T-cell lymphoma of groin	Malignant

J8	152	65	F	Lymph node	T-cell lymphoma of post occipitalia	Malignant
J9	153	44	M	Lymph node	Peripheral T-cell lymphoma of left groin	Malignant
J10	154	19	M	Lymph node	T-cell lymphoma of right neck	Malignant
J11	155	30	M	Lymph node	Lymphoid epithelioid T-cell lymphoma of right armpit	Malignant
J12	156	39	F	Lymph node	T-cell lymphoma of right neck	Malignant
J13	157	32	M	Lymph node	T-cell lymphoma	Malignant
J14	158	64	M	Lymph node	T lymphoblastic lymphoma of right groin	Malignant
J15	159	60	F	Lymph node	T-cell lymphoma of left neck	Malignant
J16	160	57	M	Lymph node	T-cell lymphoma of neck	Malignant
K1	161	42	F	Lymph node	Angioimmunoblastic T-cell lymphoma of neck	Malignant
K2	162	49	M	Lymph node	Angioimmunoblastic T-cell lymphoma of neck	Malignant
K3	163	51	M	Lymph node	Angioimmunoblastic T-cell lymphoma of groin	Malignant
K4	164	62	M	Lymph node	Angioimmunoblastic T-cell lymphoma of right groin	Malignant
K5	165	25	M	Lymph node	Nodular sclerosis Hodgkin‘s lymphoma of neck	Malignant
K6	166	4	M	Lymph node	Mixed Hodgkin‘s lymphoma of right neck	Malignant
K7	167	3	M	Lymph node	Mixed Hodgkin‘s lymphoma of right neck	Malignant
K8	168	24	M	Lymph node	Mixed Hodgkin‘s lymphoma of neck	Malignant
K9	169	65	F	Lymph node	Nodular sclerosing Hodgkin‘s lymphoma of left groin	Malignant
K10	170	18	M	Lymph node	Mixed Hodgkin‘s lymphoma of right armpit	Malignant
K11	171	37	F	Lymph node	Nodular Hodgkin‘s lymphoma of neck	Malignant
K12	172	27	M	Lymph node	Mixed Hodgkin‘s lymphoma of neck	Malignant
K13	173	33	F	Lymph node	Lymphocyte predominant Hodgkin‘s lymphoma of left neck	Malignant
K14	174	36	M	Lymph node	Lymphocyte predominant Hodgkin‘s lymphoma of right neck	Malignant
K15	175	40	F	Lymph node	Mixed Hodgkin‘s lymphoma of right neck	Malignant
K16	176	42	M	Lymph node	Lymphocyte predominant Hodgkin‘s lymphoma of left neck	Malignant
L1	177	21	F	Lymph node	Lymph node tissue	Normal
L2	178	30	M	Lymph node	Lymph node tissue	Normal
L3	179	18	F	Lymph node	Lymph node tissue	Normal
L4	180	32	M	Lymph node	Lymph node tissue	Normal
L5	181	31	M	Lymph node	Lymph node tissue	Normal
L6	182	35	M	Lymph node	Lymph node tissue	Normal
L7	183	21	F	Lymph node	Lymph node tissue	Normal
L8	184	35	M	Lymph node	Lymph node tissue	Normal
L9	185	38	M	Lymph node	Lymph node tissue	Normal
L10	186	45	M	Lymph node	Lymph node tissue	Normal
L11	187	42	M	Lymph node	Lymph node tissue	Normal
L12	188	35	M	Lymph node	Lymph node tissue	Normal
L13	189	30	M	Lymph node	Lymph node tissue	Normal

L14	190	34	M	Lymph node	Lymph node tissue	Normal
L15	191	30	M	Lymph node	Lymph node tissue	Normal
L16	192	35	M	Lymph node	Lymph node tissue	Normal
-	0	42	M	Adrenal gland	Pheochromocytoma (tissue marker)	Malignant
